# The Year Book of Treatment for 1891

**Published:** 1891-05

**Authors:** 


THE YEAR-BOOK OF TREATMENT FOR 1891. A Critical Review for Practitioners of Medicine and Surgery. Philadelphia: Lea Brothers & Co. 1891.

This annual, which is published in duodecimo form and contains 480 pages, is one of the best reference books that comes to the hands of the physician. The work of the twenty contributors is exceptionally good, and we think the volume admirably fills the place it is designed to occupy. It is a pleasure to have, once in awhile, a book published for practitioners especially, and we think those who are fortunate enough to possess this one ought to appreciate the enterprise of the publishers in serving the profession so admirably.

